# Achaete-Scute Complex Homolog-1 Promotes DNA Repair in the Lung Carcinogenesis through Matrix Metalloproteinase-7 and O(6)-Methylguanine-DNA Methyltransferase

**DOI:** 10.1371/journal.pone.0052832

**Published:** 2012-12-26

**Authors:** Xiao-Yang Wang, Sandra M. Jensen-Taubman, Kathleen M. Keefe, Danlei Yang, R. Ilona Linnoila

**Affiliations:** Cell and Cancer Biology Branch, Center for Cancer Research, National Cancer Institute, National Institutes of Health, Bethesda, Maryland, United States of America; H. Lee Moffitt Cancer Center & Research Institute, United States of America

## Abstract

Lung cancer is the leading cause of cancer-related deaths in the world. Achaete-scute complex homolog-1 (Ascl1) is a member of the basic helix-loop-helix (bHLH) transcription factor family that has multiple functions in the normal and neoplastic lung such as the regulation of neuroendocrine differentiation, prevention of apoptosis and promotion of tumor–initiating cells. We now show that Ascl1 directly regulates matrix metalloproteinase-7 (MMP-7) and O(6)-methylguanine-DNA methyltransferase (MGMT). Loss- and gain-of-function experiments in human bronchial epithelial and lung carcinoma cell lines revealed that Ascl1, MMP-7 and MGMT are able to protect cells from the tobacco-specific nitrosamine NNK-induced DNA damage and the alkylating agent cisplatin-induced apoptosis. We also examined the role of Ascl1 in NNK-induced lung tumorigenesis *in vivo*. Using transgenic mice which constitutively expressed human Ascl1 in airway lining cells, we found that there was a delay in lung tumorigenesis. We conclude that Ascl1 potentially enhances DNA repair through activation of MMP-7 and MGMT which may impact lung carcinogenesis and chemoresistance. The study has uncovered a novel and unexpected function of Ascl1 which will contribute to better understanding of lung carcinogenesis and the broad implications of transcription factors in tobacco-related carcinogenesis.

## Introduction

Lung cancer is the leading cause of cancer-related deaths in the world [1], [2]. It is estimated that there will be nearly 226,160 new cases and 160,340 deaths from lung cancer in the United States in the year 2012 [3]. The major clinical categories are non small-cell lung cancer (NSCLC), which represents the majority and small cell lung cancer (SCLC). Based on neuroendocrine (NE) features, lung cancers can also be divided into NE and non-NE tumors. Up to 25–30% of lung cancers may have NE features, which include the prototypic NE cancer SCLC [4], [5].

Achaete-scute complex homologue-1 (Ascl1) [6] is a member of the basic helix-loop-helix (bHLH) transcription factor family, which activates transcription by binding to the E-box (5′-CANNTG-3′) in the promoter region. Ascl1 is essential for NE cell development and differentiation in the lung and SCLC [6], [7]. Moreover, in subsequent studies we have shown that Ascl1 has multiple functions in a variety of other cell types [7], [8], [9], [10](). For instance, constitutive expression of Ascl1 in mouse airway epithelium promotes proliferation and resistance to apoptosis in non-NE cells, resulting in potentially premalignat lesions called bronchiolization of the alveoli (BOA) [8], [9]. We discovered that BOA lesions contained increased levels of matrix metalloproteinase-7 (MMP-7) lesions [11]. When MMP-7 was expressed in human lung cancer or immortalized airway epithelial cells, it enhanced growth and migration. Interestingly, one of the differentially expressed genes in the cells was O(6)-methylguanine-DNA methyltransferase (MGMT) [11].

Smoking is the most widely established risk factor for lung carcinogenesis. It has been reported that tobacco use is the main cause in 90% of lung cancer deaths [12], [13]. Although many factors in tobacco smoke contribute to lung cancer, nitrosamine 4-(methylnitros-amino)-1-(3-pyridyl)-1-butanone (NNK, nicotine-derived nitrosamine ketone) is one of the ingredients which plays a major role in lung carcinogenesis [14] and is widely used in lung cancer research. In the current study we focused on the MGMT gene which encodes the drug metabolizing enzyme O6-alkylguanine DNA alkyltransferase and its potential role in Ascl1-mediated tobacco-related lung carcinogenesis. Using immunohistochemistry (IHC) we showed that most human SCLCs were positive for MGMT. It is one of the genes participating in the repair of DNA adducts caused by tobacco-related carcinogens and chemotherapy [15], [16]. This will be important for the pathobiology of SCLC which typically expresses Ascl1 and is initially responsive to cytotoxic treatment but rapidly develops resistance. Through transfection and RNAi techniques, we studied the potential molecular pathways involved by MGMT and Ascl1 in human lung epithelial cells *in vitro*. Moreover, we used transgenic mice to investigate the impact of Ascl1 on tumorigenesis *in vivo* after the exposure to NNK. The results suggest that activation of MMP-7 and MGMT by Ascl1 may contribute to a delay in tobacco-related lung carcinogenesis in mice. The findings illustrate yet another novel and potentially important function of Ascl1in lung carcinogenesis which is not limited to NE phenotype.

## Materials and Methods

### Cell lines, gene expression and reporter assays

The human SCLC line DMS53 (ATCC, Manassas, VA) was grown in Waymouth's medium (Invitrogen, Rockville, MD, USA) supplemented with 15% fetal bovine serum (FBS), 100 units of penicillin and 100 µg of streptomycin per ml. The peripheral adenocarcinoma cell line NCI-H441 (H441, ATCC, Manassas, VA) was grown in RPMI 1640 (Invitrogen) supplemented with 10% FBS (Invitrogen) and antibiotics at 37°C and 5% CO_2_. The immortalized human bronchial epithelial BEAS-2B cells (ATCC) were cultured in bronchial epithelial cell growth medium with ‘bullet kit’ additives (Cambrex Bio-science, Walkersville, MD, USA) composed of growth factors and antibiotics. The establishment of stable Ascl1 or MMP-7 expression in the cells has been previously described [7], [8], [11]. The expression of each gene was tested by quantitative real-time PCR (qRT-PCR) and IHC. The mouse lung adenocarcinoma line, CL-13 [17] was a gift from Dr. Steven Belinski (Lovelace Respiratory Research Institute, Albuquerque, NM). CL-13 was cultured using RPMI 1640 (Invitrogen) supplemented with 10% FBS (Invitrogen) and antibiotics at 37°C and 5% CO_2_.

To confirm the impact of pro-MMP7 on MGMT expression, a full-length human MMP-7 sequence (with intact prodomain and functionally active) was cloned into the pCMV6-AC-GFP vector (pCMV6-AC-GFP-MMP7). A constitutively expressed active MMP-7 construct (pCMV6-AC-GFP-MMP7/Pro(−)) which was created by removing nucleotides 64–282, corresponding to the inhibitory or mutant prodomain (Pro(−), amino residues 2–94) [18]. The full-length MMP-7 and mutant (deleted) pro-MMP-7 domain sequence in vector pCMV6-AC-GFP were verified by sequencing. BEAS-2B cells were transfected with pCMV6-AC-GFP-MMP-7, pCMV6-AC-GFP-MMP7/Pro(−) or empty vector using Turbofectin 8.0 (OriGene, Rockville, MD). Forty-eight hours later, the cells were selected with geneticin (G418) at concentration 1500 µg/ml for 10 days. The stable expressing cells and conditional media were harvested. Media were concentrated at 50 times and analyzed by Western blot (WB) using anti-MMP-7 antibody which recognized both pro- and active-MMP-7. Cell lysates were analyzed by WB using anti-MGMT and β-actin antibodies.

MMP-7 and MGMT expression was ‘knocked down’ using a shRNAmir GIPZ lentiviral vector targeting the sequence of MMP-7 or MGMT in the 3′-UTR of MMP-7 or MGMT mRNA (Open Biosystems, Huntsville, AL). TLA-HEK293T cells (Open Biosystems) were transfected with the Trans-Lentiviral Packaging Mix and pGIPZ transfer vector at 50% confluence using Arrest-In transfection reagent (Open Biosystems) according to the manufacturer's protocol. After incubation for 48–72 hr, the virus-containing supernatant was collected and centrifuged at 3,000 rpm for 20 min at 4°C, mixed 50∶50 with fresh cell culture media, and used to transduce BEAS-2B/MMP-7 and H441/Ascl1 cells. Lentivirus expressing a non-silencing control shRNA (shRNAmir, Open Biosystems) served as a negative control. Cells were selected for stable integration of the virus by incubation with 2.5 µg/ml puromycin (Sigma-Aldrich Corp.) for 10 days. The efficiency of integration was monitored by green fluorescent protein (GFP) co-expressed by the lentivirus.

For the luciferase reporter assays, BEAS-2B/empty vector or /MMP-7 cells were grown in 24-well plates in the regular medium. After 24 hrs the medium was replaced with serum free medium, We co-transfected MGMT luciferase reporter plasmid (pGL2-hMGMT-Luc) (a kind gift from Sankar Mitra, Department of Biochemistry & Molecular Biology, University of Texas Medical Branch) [19] and control reporter renilla luciferase vector (pRL-SV40) (Promega, Madison, WI) into the cells using Lipofectamine Plus (Invitrogen). The co-transfection ratio for pGL2-hMGMT-Luc: pRL-SV40 plasmid DNA was 1∶40. The activity of control reporter (renilla luciferase vector) provides an internal control as the baseline which minimizes experimental variability due to differences in cell viability and transfection efficiency. We normalized the activity of the MGMT reporter to that of the internal control luciferase activity which were measured with the dual-luciferase assay system (Promega) according to the manufacture's protocol.

### Quantitative real times PCR (qRT-PCR), Western blot (WB) and chromatin immunoprecipitation (ChIP)

Total RNA was extracted from cultured cells using the RNeasy Mini kit (Qiagen, Valencia, CA) following the manufacturer's protocol and qRT-PCRwas performed as previously described [20]. The PCR reactions for the target genes and 18 s rRNA (internal control) were performed in separate tubes to avoid possible competition and/or interference in a single reaction tube. Primers for qRT-PCR are listed in Table S1.

Cell lysates were prepared on ice for WB as previously described [20]. Proteins were detected with the appropriate primary antibody (see Table S2) using horseradish peroxidase-linked secondary antibodies, and visualized by chemiluminescence with the West Pico system (Amersham Biosciences, Piscataway, NJ).

Chromatin immunoprecipitation (ChIP) assay was performed using a Magna-ChIP™ A kit (Millipore, Billerica, MA) on DMS53 cells. Cross-linking was performed via 10-min incubation in 1% formaldehyde. After lysis, sonication yielded an average fragment size of 200–1000 bp. Solubilized chromatin was immunoprecipitated with mouse IgG for negative control, monoclonal antibody against Ascl1 or anti-acetyl-histone H3 (AcH3) antibody from the kit for positive control overnight at 4°C [21]. Magnetic protein A beads were added and incubated at 4°C for 2 hours. PCR was performed using primers directed at conserved regions containing the Ascl1-binding E-box motif identified in the proximal promoter regions of the MMP-7 and MGMT genes (Table S1). Anti-AcH3 antibody and GAPDH promoter primers from the kit were used as a positive control. Input represents a 10-fold dilution of unprecipitated genomic DNA.

### DNA damage and comet assay

Phosphorylated histone H2AX (Ser139) is a marker for DNA strand brake [22], [23] which we used for screening DNA damage by Western blot in H441 and BEAS-2B cells. The study of DNA damage by NNK *in vitro* is difficult due to its complex enzymatic activation. To bypass this problem, a precursor 4-[(acetoxymethyl) nitrosamino]-1-(3-pyridyl)-1-butanone (NNKOAc) is commonly used. For quantitation, cells were harvested and washed with 1× PBS after 2 hours of treatment with NNKAOc (Toronto Research Chemicals Inc, ON, Canada) or after 2 hours of treatment with NNKOAc plus 24 hours recovery. Fifty µL of cell suspension (1×10^5^/mL) was mixed with 500 µL of 0.5% low-melting-point agarose at 37°C, and 75 µL was immediately added to Comet Slides (Trevigen, Gaithersburg, MD). After hardening, slides were immersed in prechilled lysis buffer [2.5 mol/L NaCl, 100 mmol/L EDTA (pH 10), 10 mmol/L Tris, containing 1% Triton X-100 and 10% DMSO] at 4°C for 1 hr, washed with distilled water and placed horizontally in a gel chamber. They were submerged in freshly made alkaline buffer [300 mmol/L NaOH/1 mmol/L EDTA (pH 13)] for 40 minutes followed by electrophoresis at 25 V (0.86 V/cm), 300 mA for 25 minutes at 4°C to detect both single- and double-stranded DNA breaks. Slides were then neutralized in 0.4 mmol/L Tris-HCl (pH 7.4), rinsed with distilled water, placed in prechilled 70% ethanol for 5 minutes, and air dried overnight at room temperature. After staining with SYBR Green (Trevigen), comets were assessed by microscopy (×10 objective) using a Leica DMI 4000B microscope equipped with a digital camera (Qimaging, Surrey, BC Canada) and analyzed using the TriTek CometScore® software (TriTek Corporation, Sumerduck, VA). The percentage of DNA in the tail (tail DNA %) and tail moment (% of DNA in the tail×tail length) was determined for 50 comets in each sample.

### Cytotoxicity assay

Cis-diamminedichloroplatinum (II) (cisplatin or DDP) (Sigma, St. Louis,MO) was dissolved in 0.9% sodium chloride (NaCl). Etoposide (Sigma) and docetaxel (Sigma) were dissolved in dimethyl sulfoxide (DMSO). Cells were seeded in 96-well plates at 3,000 cells/well for BEAS-2B and 5,000 cells/well for the H441 cell line in 200 µl medium. The concentrations of incubation were 0, 0.5, 5, 50 µM for cisplatin and 0, 1, 10,100 µM for etoposide or docetaxel. The cell viability was determined by sulforhodamine B staining after 48 hr treatment with chemotherapeutic drugs [24].

### NNK experiments with mice

Mice were housed under specific pathogen-free conditions under a 12-hour light/dark cycle with access to food and water *ad libitum* according to an approved protocol by National Institutes of Health (NIH) Animal Care and Use Committee (No. CCBB004). CC10-Ascl1 transgenic FVB mice were generated as previously described and their wild-type (WT) littermates were used as controls [9]. At four months of age, mice were injected with NNK (Toronto Research Chemicals Inc, ON, Canada) (100 mg/kg, I.P.) or solvent (0.9% saline) once a month, for a total of three injections. At 24 or 52 weeks after the first injection, mice were weighed, and sacrificed for lung tissue collection, counting and measuring tumors.

### Immunohistochemistry (IHC) and morphometry

Formalin-fixed paraffin-embedded tissue sections were stained using Vectastain ABC kits (Vector Laboratories, Burlingame, CA) following the vendor's instructions with modifications as described [25]. Archival paraffin blocks of human lung cancer specimens were obtained from IRB-approved protocols at NCI, NIH. Microwave antigen retrieval in 0.01M citrate buffer (pH 6.0) was performed for slides stained with antibodies to calcitonin gene-related peptide (CGRP), protein gene product 9.5 (PGP9.5), MGMT and Ascl1. Information on antibodies appears in Table S2.

For morphometric measurements MetaMorph® image analysis software (Molecular Devices, Inc. Sunnyvale, CA) was used to capture and examine microscopic images. The expression of MGMT was quantified 1) in the most distal 100 µm of terminal bronchioles at bronchioloalveolar duct junctions, 2) BOA lesions of CC10-Ascl1 transgenic mice and 3) tumors by counting individual cells that were scored either negative or positive. Sections from 32 mice (4 in each category) were included. On average, 600∼800 cells in terminal bronchioli and BOA lesions (per each structure) and over 1000 cells per tumor in each slide were examined. The percentage of positive cells was calculated to compute the relative expression of MGMT.

### DNA isolation, bisulfite modification and PCR amplification for pyrosequencing

For the methylation assay, DNA was isolated from the lungs using the DNeasy Tissue kit (Qiagen) with the addition of proteinase K digestion steps as per manufacturer's suggestions. Bisulfite modification was done using Zymo Research EZ Methylation Gold kit (Zymo Research, Orange, CA). Total 500 ng of sample DNA was used for bisulfite modification followed by PCR amplification. Mouse MGMT CpG loci at ENSMUSG00000054612 or −242 to −118 from the exon 1 transcriptional start site were amplified. Unmethylated DNA control and *in vitro* methylated DNA were mixed at different ratios followed by bisulfite modification, PCR and pyrosequencing analysis by EpigenDx Inc (Worcester, MA). The percent of methylation was correlated with expected methylation percentages with an r-square of 0.8 or higher. The PSQ™96HS system (Qiagen) was used according to standard procedures with the dispensation orders assigned for each assay. Methylation index for the MGMT promoter was calculated as the mean value of mC/(mC+C), where C is unmethylated cytosine and mC is 5′ methyl-cytosine, for all examined CpGs in the target sequence.

### Statistical Analysis

SPSS software version 16 (SPSS Inc Chicago, IL, USA.) was used for all statistical analysis. Results are expressed as the mean±SEM. Pearson chi-square test or Fisher exact test was used to compare the frequency of lung tumors. Two-tailed Student's *t* test was used to analyze the difference between two groups. One-way ANOVA analysis was used for comparing the difference among more than two groups. A *P* value less than 0.05 was considered statistically significant.

## Results

### MGMT is expressed in human airways and SCLCs

**Figure 1:**
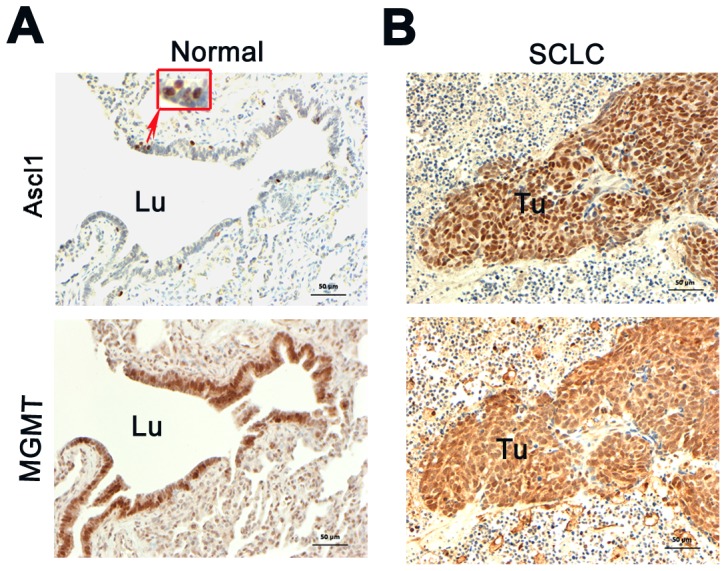
Expression of Ascl1 and MGMT in human airways and SCLCs. A) Normal airways contained only scattered Ascl1 positive NE cells, while most of the epithelium was positive for MGMT (lower panel). B) Adjacent sections of SCLC with sheets of tumor cells positive for Ascl1 (upper panel) and MGMT (lower panel). Immunoreactive tumor areas were surrounded by negative stromal tissue. Note the predominantly nuclear distribution of immunoreativity for both Ascl1 and MGMT. (Immunoperoxidase staining, bar = 50 µm). Lu = lumen, Tu = tumor.

We investigated the expression of MGMT in human lung by IHC in 14 specimens of normal airways and 16 lung NE cancers, including 15 SCLCs and one large cell NE lung carcinoma. As expected, a few scattered Ascl1 positive cells were located in airway epithelium consistent which the low number of pulmonary NE cells while all NE carcinomas were Ascl1 positive. Almost all of the airway epithelial cells were MGMT positive (Fig. 1A). We also found moderate to strong nuclear MGMT immunoreactivity in 12 out of 15 (80%) SCLCs, while the single large cell NE carcinoma was negative. At times, intense nuclear expression of MGMT was also noted in the surrounding stromal tissues. The staining results indicate that MGMT is widely expressed in human SCLCs.

### Ascl1 upregulates MMP-7 and MGMT expression through binding to the E-box in their promoters

**Figure 2:**
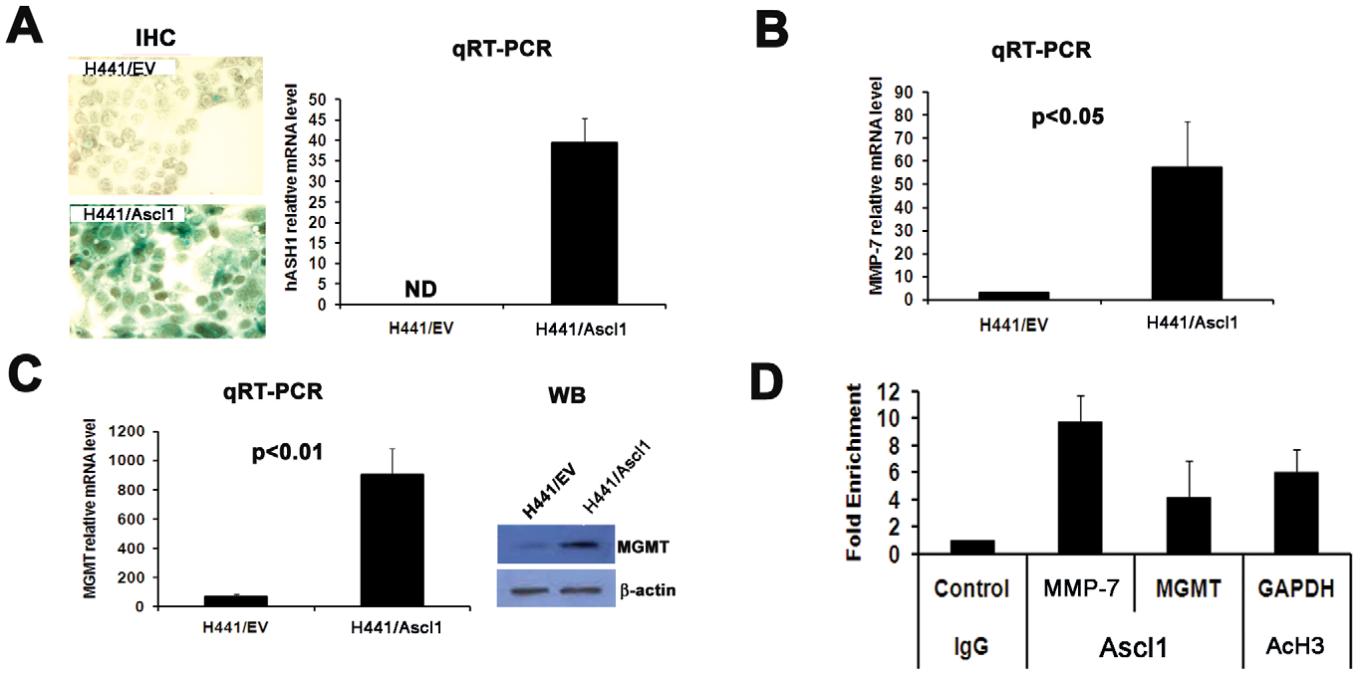
MMP-7 and MGMT are downstream from Ascl1. A) Overexpression of Ascl1 in the human lung adenocarcinoma cell line H441. Photomicrographs of Ascl1 expression by IHC (left panels –immunoperoxidase stain). Ascl1 mRNA expression (Right panel - qRT-PCR). B) Increased MMP-7 mRNA expression in H441/Ascl1 cells (qRT-PCR. C) Increased expression of MGMT mRNA (left panel – qRT-PCR) and protein (right panel - WB) in H441/Ascl1 cells. D) ChIP assay of the human SCLC DMS53 cell line demonstrated that Ascl1 directly binds to both MMP-7 and MGMT promoter DNAs. IgG was used as a negative and anti-AcH3 antibody and GAPDH promoter region primers as a positive control. EV = empty vector.

To investigate if MMP-7 and MGMT are downstream genes of Ascl1, we overexpressed Ascl1 in human lung adenocarcinoma cells H441 which are normally negative for Ascl1 expression (Fig. 2A). We showed increased expression of both MMP-7 and MGMT in Ascl1-overexpressing H441 cells. (Fig. 2B–C). Since Ascl1 is a bHLH transcription factor, it may directly activate these downstream genes by binding to the E-box of in the promoters. To test this possibility, we used an Ascl1 positive SCLC cell line DMS53 in a chromatin immunoprecipitation (ChIP) assay to determine if Ascl1 protein is able to bind to MMP-7 and MGMT promoters. Our results showed that the MMP-7 promoter had a ten- fold and the MGMT promoter had over four- fold enrichment of DNA copies bound to the Ascl1 protein in DMS53 cells (Fig. 2D). This suggests that MMP-7 and MGMT are direct targets of Ascl1 gene *in vitro*.

### Expression of MMP-7 in airway cells enhances MGMT activity

**Figure 3:**
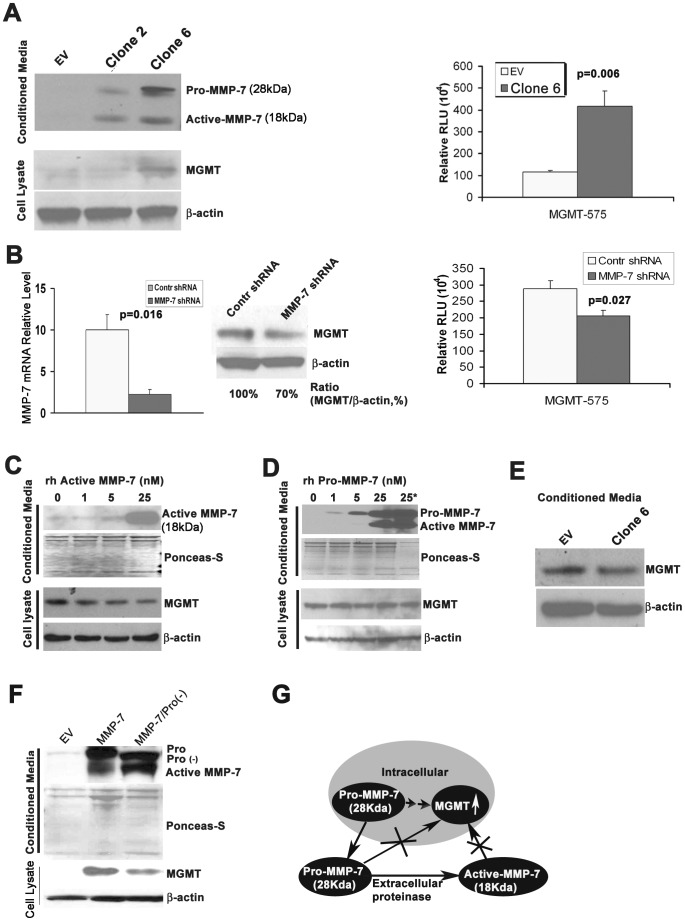
Overexpression of MMP-7 in human bronchial epithelial cells leads to increased levels MGMT. A) Overexpression of MMP-7 in the immortalized human bronchial epithelial BEAS-2B cells by stable transfection increased MGMT protein (left panel – WB) and promoter activity (mean±S.E.M p<0.001, right panel). EV = BEAS-2B/empty vector (control); Clone 2 = BEAS-2B/MMP-7 clone 2 with low expression of MMP-7; Clone 6 = BEAS-2B/MMP-7 clone 6 with high expression of MMP-7. B) Knockdown of MMP-7 mRNA in Clone 6 (left panel) decreased MGMT expression (middle panel) and promoter activity (p<0.05, mean±S.E.M, right panel). C) The expression of MGMT in BEAS-2B cells following treatment by recombinant human active MMP-7 (rh MMP-7) for 48 hours. There was no increase in MGMT expression (WB). D) The expression of MGMT in BEAS-2B cells following treatment by recombinant human pro-MMP-7 (rh pro-MMP-7) for 48 hours. There was no increase in MGMT expression (WB). E) The expression of MGMT in parent BEAS-2B cells following treatment by conditioned media from Clone 6 cells for 48 hours. There was no change in MGMT expression. F) Constitutive expression of MMP-7 without pro-domain caused lower expression of MGMT than the overexpression of full length MMP-7 with intact pro-domain in BEAS-2B cells. The mutant MMP-7 band indicated by Pro(−) in the left lane migrates faster than the full length pro-MMP-7 (Pro) in the middle lane (WB). G) Proposed model for the regulation of MGMT: MMP-7 is synthesized in the cells as pro-MMP-7 which is associated with increased levels of MGMT (double arrow) by an unknown mechanism. It is likely to be one of many factors that contribute to increased MGMT expression. Pro-MMP7 is secreted and cleaved by proteases to its active form outside the cell (arrow). We have shown that neither extracellular pro-MMP-7 nor active MMP-7 has any impact on MGMT (crossed arrows).

We have shown [11] that overexpression of MMP-7 in the immortalized human normal airway epithelial BEAS-2B cells increases MGMT expression. To demonstrate that the expression also correlates with activity, we now performed a MGMT luciferase reporter assay. MGMT promoter activity was dramatically increased in MMP-7-overexpressing BEAS-2B cells (Fig. 3A). Furthermore, knockdown of MMP-7 in the cells resulted in a 30% decrease of MGMT protein expression and a significant decrease in its promoter activity (Fig. 3B). The results suggest MGMT is a downstream from MMP-7.

The matrix metalloproteinase MMP-7 is a secretory protein that has an active form (18 kDa) and a pro-form (28 kDa). To investigate which form is important for the regulation of MGMT expression, BEAS-2B cells were incubated with recombinant human active MMP-7, pro-MMP-7, or conditioned media from BEAS-2B/MMP-7 overexpressing cells (Fig. 3C–E). The expression of MGMT remained unchanged. To address the lack of response by MGMT, the pro-domain was removed. As a result, constitutive expression of active MMP-7 without the pro-domain sequence decreased MGMT levels in BEAS-2B cells (Fig. 3F) when compared to the levels of expression associated with the full length MMP-7 with intact prodomain. Based on these data, we conclude that the expression of MGMT is mediated by a currently unknown mechanism (Fig. 3G) which may indirectly involve MMP-7.

### MGMT protects cells from tobacco-related DNA damage

DNA damage is an important early step in tobacco-related carcinogenesis. NNK, like many other carcinogens, is metabolized to its chemically active form in the lung by Clara cells and in the liver by hepatocytes. In order to explore the role of MGMT in the repair of DNA damage *in vitro* we turned to the chemically active metabolite NNKOAc of the tobacco-specific nitrosamnine NNK. NNKOAc generates damage at all four bases with decreasing order; guanine>adenine>cytosine>thymine and is commonly used for *in vitro* experiments [26].

**Figure 4:**
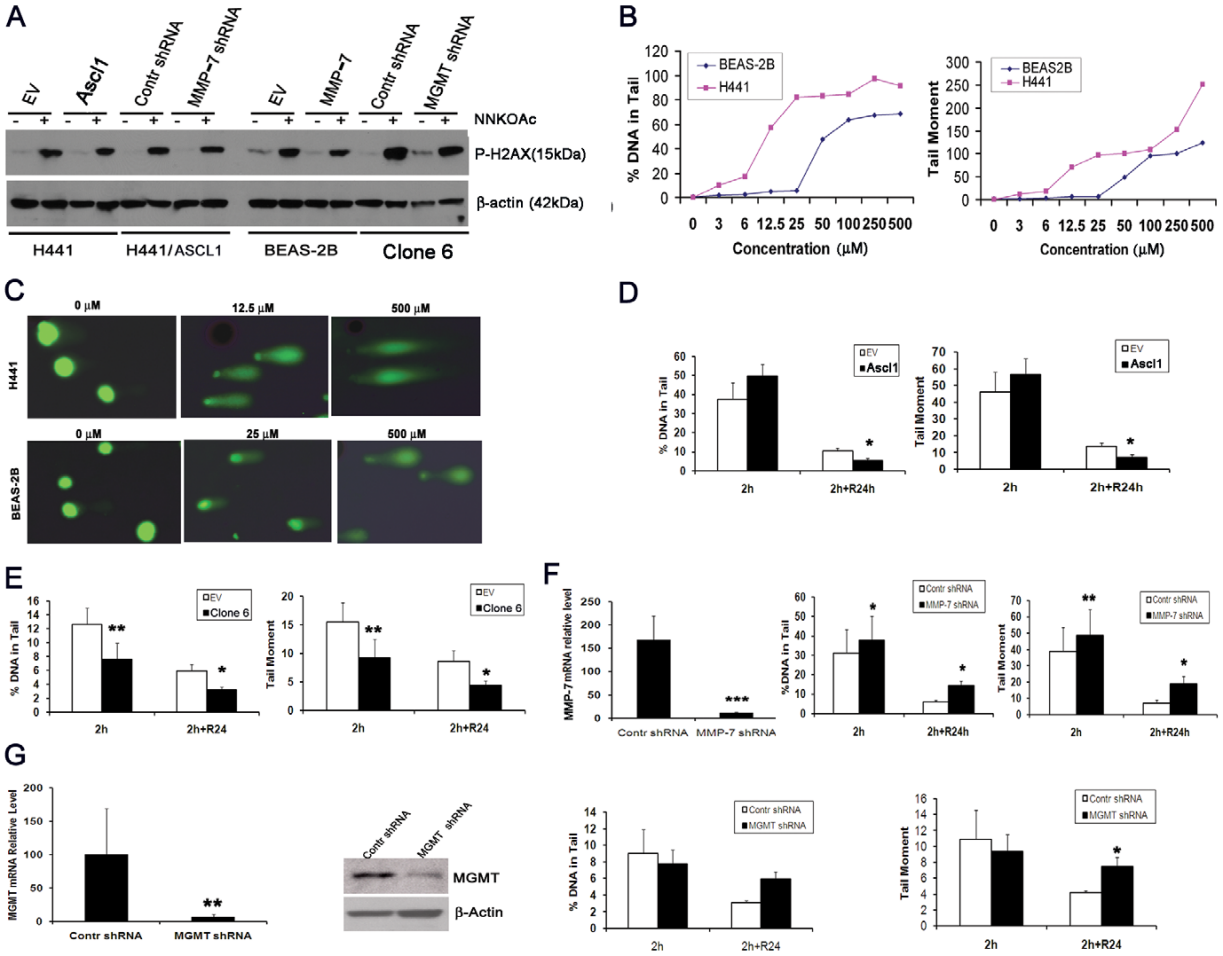
Ascl1 and MMP-7 protect cells from DNA damage *in vitro*. A) Induction of phosphorylated H2AX expression by NNKOAc. Control and transfected cell lines were incubated for 2 hours with the active NNK metabolite NNKOAc and subjected for WB analysis B) Dose response curves of DNA damage in H441 and BEAS-2B cells measured by comet assay after the exposure to NNKOac at various concentrations. Two indices of DNA damage (%DNA in tail and tail moment) were used. H441 cells were more sensitive to NNKOAc treatment than BEAS-2B cells. C) Illustration of comet shapes after the electrophoresis at different concentrations of NNKOAc. D) Overexpression of Ascl1 in H441 cells promoted the recovery of %DNA in tail and tail moment. E) Overexpression of MMP-7 in BEAS-2B not only provided resistance to NNKOAc-induced DNA damage, but also drove the recovery of DNA damage. F) Knockdown of MMP-7 in H441/Ascl1 cells increased NNKOAc-induced DNA damage and delayed recovery. G) Knockdown of MGMT in BEAS-2B/MMP-7 cells (left panels –qRT-PCR and WB) did not reveal any changes in the DNA damage, but significantly prevented the recovery (right panels). At least 50 cells were analyzed in each comet experiment. (Mean ± S.E.M. in three or four independent experiments).

We used blot to demonstrate the expression of the DNA damage marker phosphorylated histone H2AX. It was expressed in the human adenocarcinoma H441 cells treated by NNKOAc at 6.25 µM and in the BEAS-2B cells treated at 12.5 µM for 6 days (Fig. 4A). The results indicate that NNKOAc causes DNA damage in these cells.

Another well established method for the detection of DNA damage is comet assay. To investigate whether Ascl1 might play a role through its downstream effectors in tobacco-related DNA damage, H441 and BEAS-2B cells were treated with NNKOAc. Both the percent (%) of DNA in tail and tail moment (% of DNA in the tail×tail length) were used for quantification of DNA damage with the TriTek CometScore® software. To optimize the assay we tested different concentrations. The damage was dose-dependent and started at 3 µM in H441 cells and 6 µM in BEAS-2B cells. (Fig. 4B, 4C). We chose to treat the cells with 12.5 and 25 µM of NNKOAc for 2 hours, respectively, followed by no or 24 hour recovery.. The tests included H441/Ascl1 (overexpressing Ascl1), BEAS-2B/MMP-7 (overexpressing MMP-7), MMP-7 knockdown (KD) in H441/Ascl1 cells and MGMT KD in BEAS-2B/MMP-7 cells. The indeces of % DNA in tail and tail moment that measure DNA damage were approximately 50% lower in H441/Ascl1 cells than those in H441/empty vector cells (p<0.05). As there was no difference after 2 hours the data suggest that overexpression of Ascl1 in H441 cells improves the recovery but not resistance to the damage (Fig. 4D). Overexpression of MMP-7 did not only impart resistance to NNKOAc-induced DNA damage (∼40% decrease of both % DNA tail and tail moment), but also promoted recovery (∼50% decrease of both % DNA tail and tail moment following 24 hour recovery) in BEAS-2B/MMP-7 cells (Fig. 4E). To show that this is reversed when MMP-7 is knocked down, we used stable expressing shRNA against MMP-7 in H441/Ascl1 cells which resulted in 20% increase of both % DNA tail (p<0.05) and tail moment (p<0.01) at 2 hours, followed by a further increase after recovery(p<0.05). (Fig. 4F). The knockdown of MGMT by specific shRNA in BEAS-2B/MMP-7 cells delayed recovery of DNA damage (Fig. 4G).

### MGMT protects cells from cisplatin-induced apoptosis

**Figure 5:**
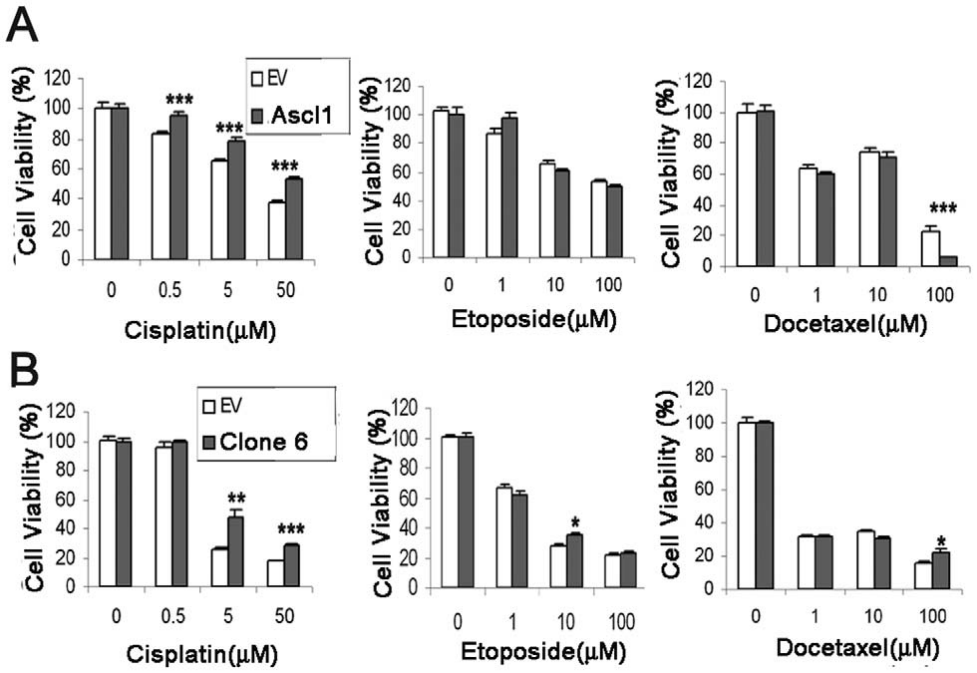
Overexpression of Ascl1 and MMP-7 makes cells resistant to cisplatin-induced cytotoxicity. A) Cell survival of lung cancer cells following treatment with chemotherapeutic drugs. H441 lung cancer cells expressing empty vector (EV) or Ascl1 were treated with cisplatin, etoposide and docetaxel at different concentrations for 48 hours. Cell viability was significantly higher in H441/Ascl1 cells after exposure to cisplatin (left panel), but there was no difference after treatment with etoposide (middle panel). When treated with docetaxel at high concentrations (100 µM), the viability was dramatically lower in H441/Ascl1 cells (right panel). B) Survival of human bronchial epithelial cells following treatment with chemotherapeutic drugs. BEAS-2B cells expressing EV or MMP-7 were treated with cisplatin, etoposide and docetaxel at different concentrations for 48 hours. The viability increased in BEAS-2B/MMP-7 cells after exposure to cisplatin at concentrations of 5 and 50 µM (left panel). The viability of BEAS-2B/MMP-7 cells was slightly higher when treated with etoposide at 10 µM., When treated with docetaxel at high concentrations (100 µM) the viability was significantly increased in BEAS-2B/MMP-7 cells (right panel). EV = empty vector (mean±S.E.M. n = 10. * P<0.05, ** p<0.01, *** p<0.001, compared with EV transfected cells with same treatment).

MGMT is a DNA repair enzyme that rapidly removes adducts at the O^6^-position of guanine [27]. Alkylating agents are known to generate a complex spectrum of adducts at the O^6^-position. To test the function of MGMT in Ascl1- and MMP-7-overexpressing cells, three first-line chemotherapeutic drugs for lung cancer treatment including cisplatin, etoposide and docetaxel were used to study sensitivity to alkylating agent-induced cell death. Cisplatin is classified as an alkylating agent, while etoposide is a topoisomerase-II inhibitor and docetaxel is a well established anti-mitotic agent. Different concentrations of cisplatin (0, 0.5, 5, and 50 µM) and etoposide or docetaxel (0, 1, 10, or 100 µM) were used for H441/Ascl1 and BEAS-2B/MMP-7 cells. Overexpression of both Ascl1 and MMP-7 caused significant resistance to cisplatin-induced cell death, suggesting that upregulation of MGMT by Ascl1 and MMP-7 in cells plays an important role in providing resistance to alkylating agents that induce cell death *in vitro* (Fig. 5).

### Upregulation of MGMT delays tobacco-related tumorigenesis in mice

**Figure 6:**
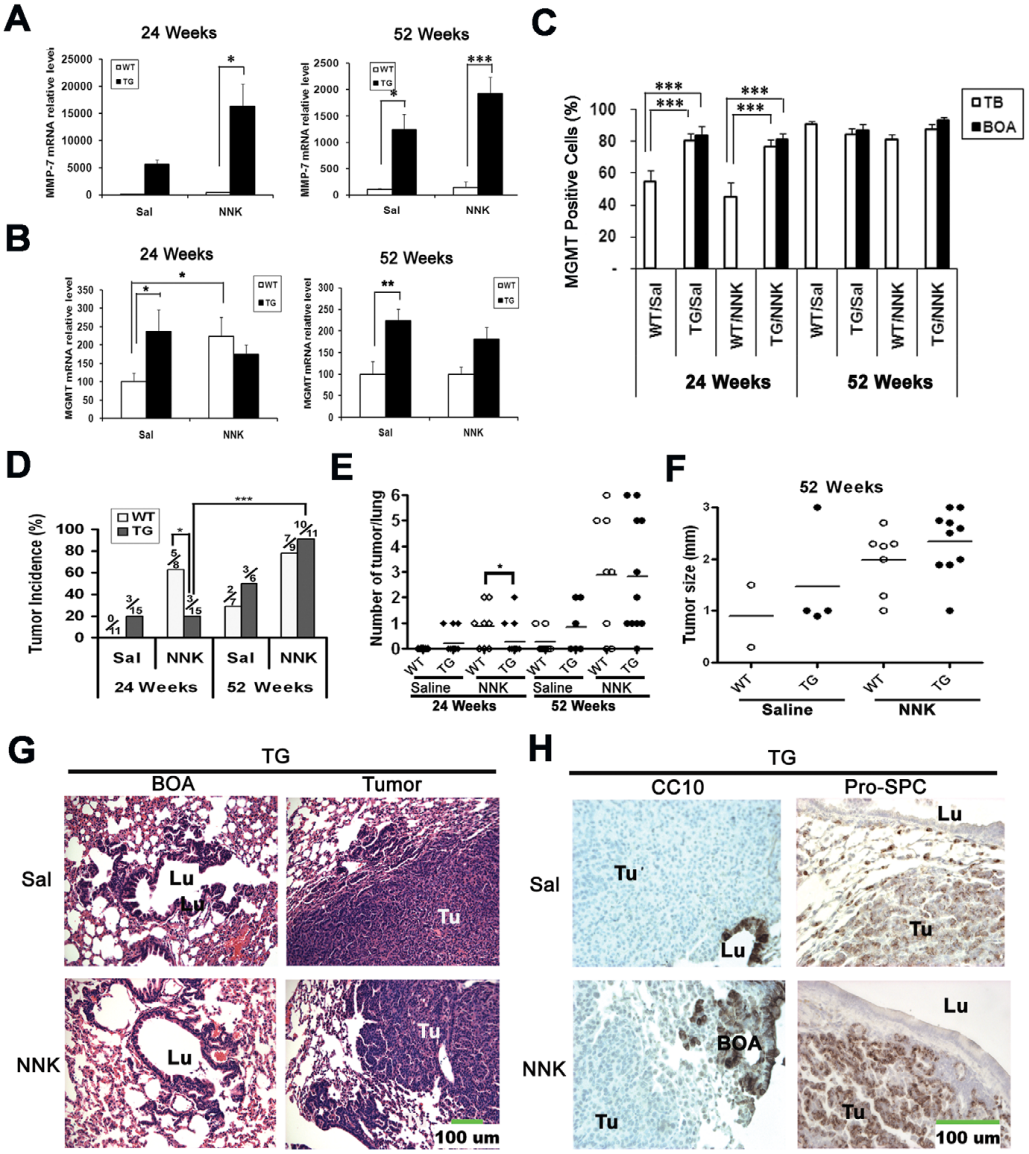
Constitutive expression of Ascl1 in the lung delays tobacco-related tumorigenesis via MGMT. A) MMP-7 mRNA was increased in the lungs of transgenic mice (qRT-PCR). At the 24 week time point, MMP-7 mRNA was detected in only 2 out of 8 WT/Sal mice and 6 out of 8 mice in WT/NNK. B) MGMT mRNA was significantly increased in the lungs of transgenic mice, but revealed no change following the exposure of these mice to NNK (qRT-PCR, n = 6∼11). C) The relative number (%) of MGMT positive cells (Fig. S1C) at terminal bronchioles and BOA lesions was increased at 24 weeks in transgenic mice regardless of NNK exposure. D) At 24 weeks the incidence of lung tumors in NNK-exposed transgenic mice was lower than that of WT mice (20% *vs* 63%; p<0.05). E) At 24 weeks the number of tumors per mouse (multiplicity) was lower in TG/NNK mice than that in WT/NNK mice. F) The size (diameter in millimiters) of surface tumors in both genotypes was comparable and somewhat larger in NNK-exposed mice. G) At 52 weeks both spontaneous and NNK-induced lung tumors in TG mice were alveolar adenomas. E) F) G) Photomicrographs of lung and tumors in transgenic mice at 52 weeks. Both spontaneous and NNK-induced tumors (Tu) were adenomas that grew independent of BOA lesions characteristic of transgenic mouse lungs (H&E staining, green bar = 100 µm). H) Photomicrographs of spontaneous and NNK-induced tumors in transgenic mice at 52 weeks. CC10 was expressed in airway epithelium (Lu) and BOA lesions, while tumors were negative. Pro-SPC was positive in normal alveolar type II cells and tumors, but negative in the epithelium lining airway lumens (Lu). (Immunoperoxidase staining, green bar = 100 µm). (Sal = saline; TG = transgenic; TB = terminal bronchiole; BOA = bronchiolization of alveoli; mean±S.E.M. * P<0.05, ** p<0.01, *** p<0.001).

We have shown that the expression of Ascl1 and MMP-7 results in increased levels and activity of MGMT *in vitro*. (Figs. 2 and 3). In order to explore the impact of MGMT on tobacco-related carcinogenesis *in vivo* we turned to transgenic CC10-hASH1 mice that constitutively expressed Ascl1 in airways under the control of CC10 promoter [8], [9]. We used a well-established and reproducible chemical carcinogenesis model where mice received the tobacco-specific nitrosamine NNK intraperitoneally and lungs were examined at 24 and 52 weeks after the first injection (Fig. 6).

To confirm the *in vitro* findings that MMP-7 and MGMT are downstream of Ascl1 gene, we determined their mRNA expression in mouse lungs using qRT-PCR. We found that both MMP-7 and MGMT mRNAs were dramatically up-regulated in transgenic mice regardless of NNK exposure (Fig. 6A, 6B). Specifically, all unexposed WT mice had much lower MGMT mRNA than transgenic ones (P<0.05 or P<0.01; Fig. 6B). Following exposure to NNK, MGMT mRNA was significantly increased in WT mice at 24 weeks, but remained low at 52 weeks (Fig. 6B).

By IHC we discovered a variable degree of MGMT nuclear immunoreactivity in the epithelia of airway and alveolar compartments of all mice (Fig. S1C). Histologically the peripheral lungs of CC10-hASH1 mice (Fig. 6G) are characterized by hyper- and dysplasias in terminal bronchioles, bronchioalveolar duct junctions and by BOA lesions [8], [9] and increased expression of MMP-7 in these areas. We now quantified MGMT positive cells in the same regions. At 24 weeks, there were significantly more positive cells in the terminal bronchioles and BOA lesions of transgenic mice regardless of the exposure to NNK (P<0.001; Fig. 6C). No difference in the percentage of MGMT positive cells was found between WT and transgenic mice at 52 weeks. The results show that there are parallels between the distributions of MGMT and MMP-7 in peripheral lungs of CC10-hASH1 transgenic animals [8], [11].

As expected, the exposure to NNK resulted in lung neoplasia. By counting the surface tumors, we found that 5 out of 8 (63%) WT mice developed pulmonary tumors, while only 3 out of 15 (20%) transgenic mice had tumors at 24weeks (p<0.05). In control mice that received saline, there were no tumors in WT mice, but 3 out 15 (20%) transgenic mice developed them at 24 weeks. At 52 weeks, 78% (7/9) of WT and 91% (10/11) of transgenic mice had tumors (p>0.05). In control mice, 29% of WT and 50% of transgenic mice had spontaneous tumors (Fig. 6D). The data indicate that constitutive expression of Ascl1 delays NNK-induced lung tumorigenesis.

At 24 weeks tumor multiplicity was higher in WT than in transgenic mice exposed to NNK (p = 0.047), while at 52 weeks there was no difference (Fig. 6E)., Regardless of the genotype, tumors in animals exposed to NNK were slightly larger than the spontaneous ones (saline groups) although the differences were not statistically significant (Fig. 6F). Histologically, all the lung tumors were adenomas and there was minimal atypia in the normal surrounding lung. Notably, tumor morphology and location in the alveolar compartment were strikingly similar in all mice, and no tumors arose from airways or BOA lesions (Fig. 6D, 6E and Fig. S1A) To further examine the properties of lung tumors, we performed IHC by using the Clara cell marker CC10 and type II cell marker surfactant protein C (Pro-SPC). As expected, pro-SPC was expressed in both NNK-induced and spontaneous lung tumors, regardless of the genotype (Fig. 6E and Fig. S1B). The expression of CC10 was limited to the airway epithelium. In addition, BOA lesions of transgenic mice were also positive for CC10, while tumors were all negative. We also examined pulmonary NE differentiation as it has been reported that both smoking in humans as well as exposure to nicotine or tobacco-specific nitrosamine NNK in animals are associated in lung NE cell hyperplasias [28]. Interestingly, quantification of PNECs and NEBs positive for CGRP revealed increased NE foci in WT but not in transgenic mice after NNK exposure. As expected, transgenic mice showed Ascl1 expression along the airway epithelium and BOA lesions that was similar in NNK-exposed and saline control animals. In contrast, Ascl1 was only expressed in rare solitary airway NE cells (PNECs) and organoid clusters called neuroepithelial bodies (NEBs) in WT mice. Notably, the two commonly used lung NE markers CGRP and PGP 9.5 were only expressed in PNECs and NEBs in both genotypes (Fig. S2). Neither Ascl1, nor the two other NE markers were expressed in NNK-induced or spontaneous lung tumors. This suggests that even in transgenic mice there is no NE differentiation in NNK-induced tumors (Fig. S2).

### Lack of MGMT methylation in the lung

Methylation is an important mechanism that may lead to the silencing of MGMT expression during lung carcinogenesis. Half of human lung cancers reveal MGMT methylation [29]. When MGMT is inactivated, it prevents DNA repair [30]. The failure to repair DNA damage can lead to mutations in genes such as *K-ras*. To explore if MGMT methylation was involved in NNK-induced lung tumorigenesis, it was analyzed by pyrosequencing. We used the mouse lung cancer cell line CL-13 as a positive control and were able to show that 66.5% of CpG islands were methylated. In contrast, there was no MGMT methylation in the lung tissues obtained from the experimental animals. This suggests that methylation of MGMT may not contribute to NNK-induced carcinogenesis in the current study (See Fig. S3).

## Discussion

In the current study we were able to show that Ascl1 directly regulates both MMP-7 and MGMT. Most of the human SCLCs that were positive for Ascl1 were also positive for MGMT by IHC. The expression of MGMT *in vitro* was associated with increased resistance to DNA damage and chemoresistance. Notably, during tobacco-related carcinogenesis in the mice that constitutively expressed Ascl1 leading to increased MMP-7 and MGMT levels, there were initially fewer tumors than in WT mice. It is plausible that the delay in tumorigenesis was caused by resistance to DNA damage. Taken together the data suggest that Ascl1 not only promotes cell growth but may also modulate NNK-induced tumorigenesis through actions of MGMT. This is in agreement with recent genome-wide studies indicating that Ascl1 is truly a multifunctional protein [31].

The tobacco-specific nitrosamine NNK is a pro-carcinogen which is first converted into its ultimate carcinogenic form that will produce DNA damage through methylation and pyridyloxobutylation adducts. The p450 enzymes mediating the conversion have been shown to be active both in Clara as well as type II cells. A variety of adducts can be isolated from smokers lungs including bulky DNA adducts that are considered to be the most dangerous ones. Likewise there are multiple repair mechanisms and failure to repair the damage may eventually lead to cancer through genomic instability, mutations, and chromosomal rearrangements [16], [17]. Such NNK-associated adducts as O6- methylguanine (O6-mG) and O6-[4-oxo-4-(3-pyridyl) butyl] guanine (O6-pobG) are removed from DNA primarily by MGMT (also referred to as O6-alkylguanine DNA alkyltransferase) through a direct reversal mechanism which restores the normal base without excision. Other mechanisms involve a pathway called nucleotide excision repair. We have previously shown that overexpression of Ascl1 increased MMP-7 expression both *in vivo* and *in vitro* and that MGMT in turn is downstream from MMP-7 [8], [11]. We therefore hypothesized that Ascl1 may activate a novel pathway that involves both MMP-7 and MGMT, resulting in DNA repair during lung carcinogenesis.

In the current study, we show that following the DNA damage *in vitro* induced by NNKOAc, an NNK derivative that does not need metabolic activation, the human H441 peripheral adenocarcinoma cells that expressed Ascl1 were quick to recover in. While the detailed mechanism for the repair of NNK-induced DNA damage is not clear, the repair by MGMT is considered to be a major pathway to remove DNA adducts [32], [33]. Notably, we show that the enforced expression of MMP-7 in human immortalized BEAS-2B bronchial epithelial cells not only prevented NNKAOc-induced DNA damage, but also enhanced DNA repair. The opposite was true after the knock-down of MMP-7. Likewise, DNA repair in the cells was delayed when MGMT was knocked down by specific RNAi. Taken together, these results provide functional evidence how both Ascl1 and MGMT may play important roles in DNA repair, and that MMP-7 is also able to regulate DNA damage and repair. This infers that delayed NNK-induced tumorigenesis in the CC10-Ascl1 TG mice may happen via promotion of DNA repair that takes place at early stages. One interpretation is that the repair activity of MGMT protein helps to decrease the probability that the damaged guanine becomes mutagenic/carcinogenic. Other studies have shown that overexpression of MGMT in transgenic mice reduces NNK-induced tumorigenesis in the lung [34]. Conversely, deficiency of MGMT dramatically increases methylnitrosourea-induced tumorigenesis in MGMT gene knockout mice [35]. In the light of these data, the fact that constitutive expression of Ascl1 significantly increased MGMT expression may contribute to the delayed NNK-induced tumorigenesis in the lungs of CC10-Ascl1 transgenic mice.

Mice overexpressing MGMT are more resistant to toxicity and tumor induction by alkylating agents [36]. To examine if alterations of the enzyme levels in human lung epithelial cells may correlate with the susceptibility to toxicity, we used two sets of human cells in which MGMT was upregulated. NCI-H441 human lung adenocarcinoma cells with forced Ascl1 expression and BEAS-2B human immortalized bronchial cells overexpressing MMP-7 were treated with the alkylating anti-cancer drug cisplatin and non-alkylating anti-cancer drugs etoposide and docetaxel. Our results showed that Ascl1 and MMP-7 were able to enhance resistance to cisplatin-induced cell toxicity. The results indicate that MGMT may function downstream from the genes by preventing alkylating agent-induced cell death. This function provides a plausible explanation why constitutive expression of Ascl1 delays *in vivo* tumorigenesis by the potent alkylating tobacco carcinogen NNK. Conversely, Ascl1 may adversely impact chemotherapy during cancer treatment by contributing to the development of chemoreristancce which commonly happens in SCLCs. Regarding human lung carcinogenesis, we found that MGMT was highly expressed in 80% of Ascl1 positive SCLCs. The data suggest that the expression of Ascl1 may enhance the resistance to alkylating chemotherapeutic drugs such as cisplatin through upregulation of MGMT in human NE lung cancers that are Ascl1 positive [37].

We discovered increased expression of MMP-7 and MGMT in the lungs of CC10-Ascl1 transgenic mice. Moreover, forced expression of Ascl1 in human peripheral adenocarcinoma NCI-H441 cells drove MMP-7 and MGMT expression. An investigation into the MMP-7 and MGMT promoters indicated that they contain several E-Box motifs. To confirm that the bHLH transcription factor Ascl1 binds to these two promoters, we performed a ChIP assay. Our results showed that Ascl1 was able to bind to both MMP-7 and MGMT promoters in the SCLC DMS53 cells that express high levels of Ascl1. Therefore, we conclude that MMP-7 and MGMT may be novel targets of the Ascl1 gene. Taken together, Ascl1 appears to have a range of targets that relate to neural, neuroendocrine as well as non-neural or epithelial cell functions [8], [10], [31].

The specific mechanism how MMP-7 interacts with MGMT was less clear. It has been reported that overexpression of MMP-7 is associated with tumor proliferation and a poor prognosis in human non-SCLCs [38]. We have previously shown that constitutive expression of Ascl1 in Clara cells of transgenic mice results in MMP-7 expression and potentially premalignant BOA lesions in the peripheral lung [8], [11]. In the current study we showed that exposure of the same transgenic mice to the potent carcinogen NNK resulted in increased MMP-7 levels, further suggesting that MMP-7 is involved in lung carcinogenesis. Our previous study indicated that enforced expression of MMP-7 in the immortalized human bronchial epithelial BEAS-2B cells leads to an increase in MGMT, suggesting that it is a downstream gene [11]. In the current paper we were able to demonstrate that the induction was associated with functional activation using the luciferase reporter assay. Conversely, knockdown of MMP-7 decreased MGMT protein levels and reporter activity.

**Figure 7:**
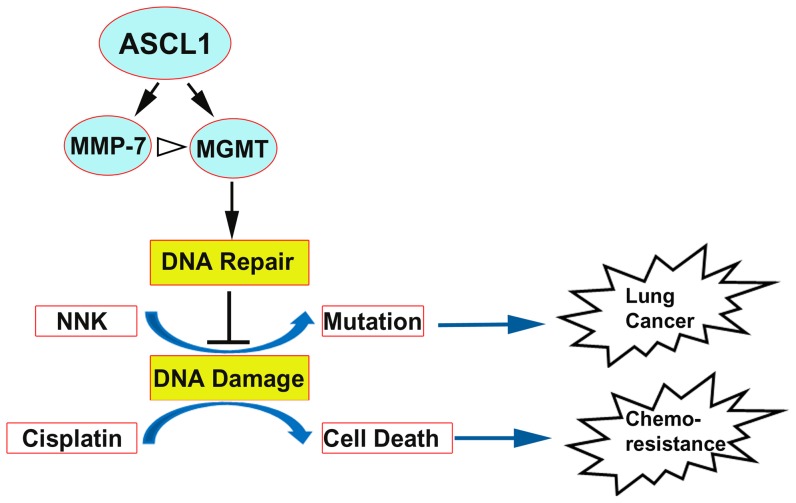
Schematic illustration how Ascl1 may modulate lung carcinogenesis and chemotherapy through MGMT expression. Ascl1 protein binds to MMP-7 and MGMT promoters, leading to increased expression (black arrows). In addition, overexpression of MMP-7 may promote MGMT expression through unknown mechanisms (white arrow head). MGMT increases DNA repair (black arrow) which attenuates DNA damage (blocked black line). This impacts tobacco-associated carcinogenesis and chemotherapy with alkylating agents. During the early phase of carcinogenesis, MGMT reduces NNK-induced DNA damage by enhancing the repair (black arrow). In turn, the increased DNA repair activity may slow down DNA damage (blocked line) associated with NNK exposure (blue arrow). During the late phase the accumulation of genetic alterations/mutations eventually leads to lung cancer. MGMT may also attenuate DNA damage (blocked line) during the alkylating agent cisplatin therapy. This impairs cell death and leads to chemoresistance (blue arrow).

In summary, we provide evidence that Ascl1 directly binds to MMP-7 and MGMT promoters, resulting in increased expression of these two genes. In addition, overexpression of MMP-7 promotes MGMT expression by an unknown mechanism. Tobacco-derived NNK is a potent carcinogen, but together Ascl1, MMP-7 and MGMT facilitate the repair of NNK-induced DNA damage and alkylating agent-induced cellular toxicity *in vitro*. Consequently, we propose that the delay seen during NNK-induced tumorigenesis in mice that constitutively express Ascl1 may involve the activation of a novel Ascl1/MMP-7/MGMT pathway (Fig. 7). In addition, MGMT activity may contribute to the development of chemoresistance in SCLCs which express Ascl1. The findings illustrate a surprising function of Ascl1, which will y be helpful for understanding early events in lung carcinogenesis. For instance, it may provide an explanation why NE cells in humans that express Ascl1 appear to be resistant to pre-malignant changes following exposure to tobacco smoke.

## Supporting Information

Figure S1 **Lung morphology and expression of the epithelial markers CC10 and Pro-SPC in WT mice and MGMT in TG mice during NNK-induced tumorigenesis.** A) Photomicrographs of the lung and pulmonary adenomas in saline- and NNK-exposed WT mice at 52 weeks (H&E staining). B) CC10 was expressed along the airways, but not in tumors. Pro-SPC was expressed in type II cells of alveoli and tumors, while airways remained negative (Immunoperoxidase staining). C) Photomicrographs of increased MGMT expression in TBs and BOAs of TG mice compared with that in the TBs of WT mice at 24 weeks. MGMT was also highly expressed in all of the lung tumors (>80%; immunoperoxidase stain). Sal = saline; Lu = lumen; Tu = tumor. TB = terminal bronciolus; BADJ = bronchioloalveolar duct junction; BOA = bronchiolization of alveoli.
(JPG)

Figure S2 **Constitutive expression of Ascl1 in Clara cells attenuates NNK-induced neuroendocrine (NE) cell differentiation in TG mice.** A) Expression of Ascl1 and CGRP in small clusters (NEBs) of NE cells (arrows) in WT mice, surrounded by negative airway epithelium and negative tumors (left panels). In TG mice, Ascl1 was expressed throughout airway epithelium and BOAs, while the NE marker CGRP was expressed in NE cells (NEB with arrow). Airway epithelium and BOA (BOA with arrow) were negative for CGRP. No Ascl1 or CGRP expression was present in tumors (bottom panels; immunoperoxidase staining). B) Bar graph of the relative numbers of CGRP-containing neuroendocrine (NE) foci (number of solitary cells plus NEBs per airways; mean±S.E.M. * P<0.05, ** p<0.01, *** p<0.001). NNK exposure increased the number of NE foci in the airways 24 weeks in WT mice, but not in TG mice. At 52 weeks, NE foci remained fewer in TG mice than in WT mice with NNK treatment. WT = wild type mice, TG = transgenic mice, Sal = saline. * p<0.05, *** p<0.001. C) A PGP9.5 positive NEB in a WT/NNK (arrow, top panel). No PGP9.5 staining in BOA (ellipse with arrow) or tumor of TG/NNK mice (bottom panel) (immunoperoxidse staining). Lu = airway lumen, Tu = tumor, BOA = bronchiolization of the alveoli, NEB = neuroepithelial body.
(JPG)

Figure S3 **Lack of MGMT methylation in the lung detected by pyrosequencing.** A) Representative pyrograms of MGMT pyrosequencing results of lungs from WT and TG mice with or without NNK treatment. Bottom two panels include a negative (UM = unmethylated) control and methylated mouse lung cancer cell line (CL-13) as a positivecontrol. B) MGMT promoter in mouse lung cancer cells CL-13 (positive control) showed methylation in 66.5% of the CpG islands, while no methylation was found in lung tissues.
(JPG)

Table S1 **Sequences of the oligonucleotide primers.**
(DOC)

Table S2 **List of Antibodies for Immunohistochemistry and Western blot.**
(DOC)
