# Supplementary figures and images for: Achaete-Scute Complex Homolog-1 Promotes DNA Repair in the Lung Carcinogenesis through Matrix Metalloproteinase-7 and O(6)-Methylguanine-DNA Methyltransferase
Source: PLoS One. 2012 Dec 26;7(12):e52832. doi: 10.1371/journal.pone.0052832 (PMC3530493; doi:10.1371/journal.pone.0052832)

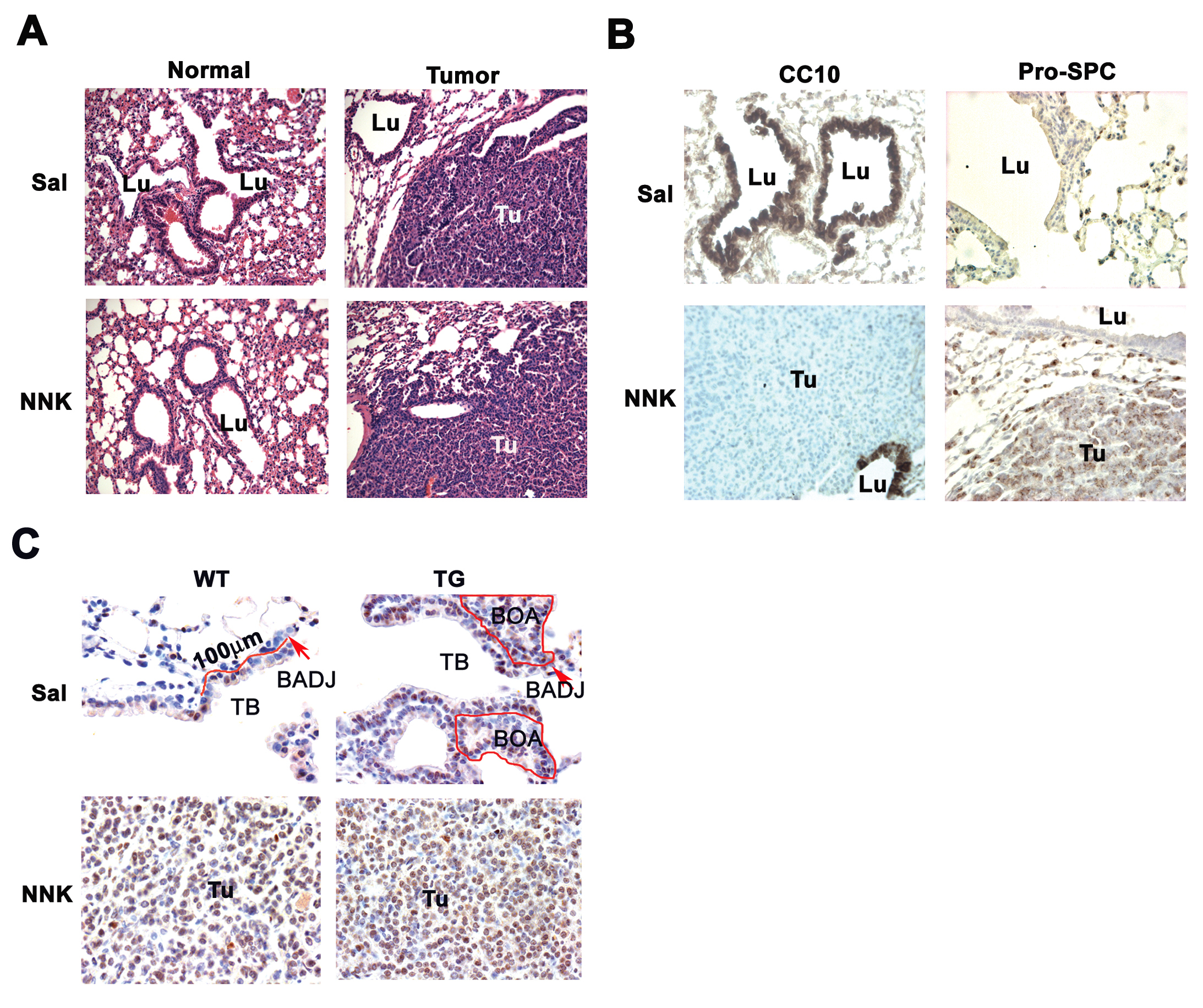

Supplement: Figure S1 — Lung morphology and expression of the epithelial markers CC10 and Pro-SPC in WT mice and MGMT in TG mice during NNK-induced tumorigenesis. A) Photomicrographs of the lung and pulmonary adenomas in saline- and NNK-exposed WT mice at 52 weeks (H&E staining). B) CC10 was expressed along the airways, but not in tumors. Pro-SPC was expressed in type II cells of alveoli and tumors, while airways remained negative (Immunoperoxidase staining). C) Photomicrographs of increased MGMT expression in TBs and BOAs of TG mice compared with that in the TBs of WT mice at 24 weeks. MGMT was also highly expressed in all of the lung tumors (>80%; immunoperoxidase stain). Sal = saline; Lu = lumen; Tu = tumor. TB = terminal bronciolus; BADJ = bronchioloalveolar duct junction; BOA = bronchiolization of alveoli. (JPG) [file pone.0052832.s001.jpg]

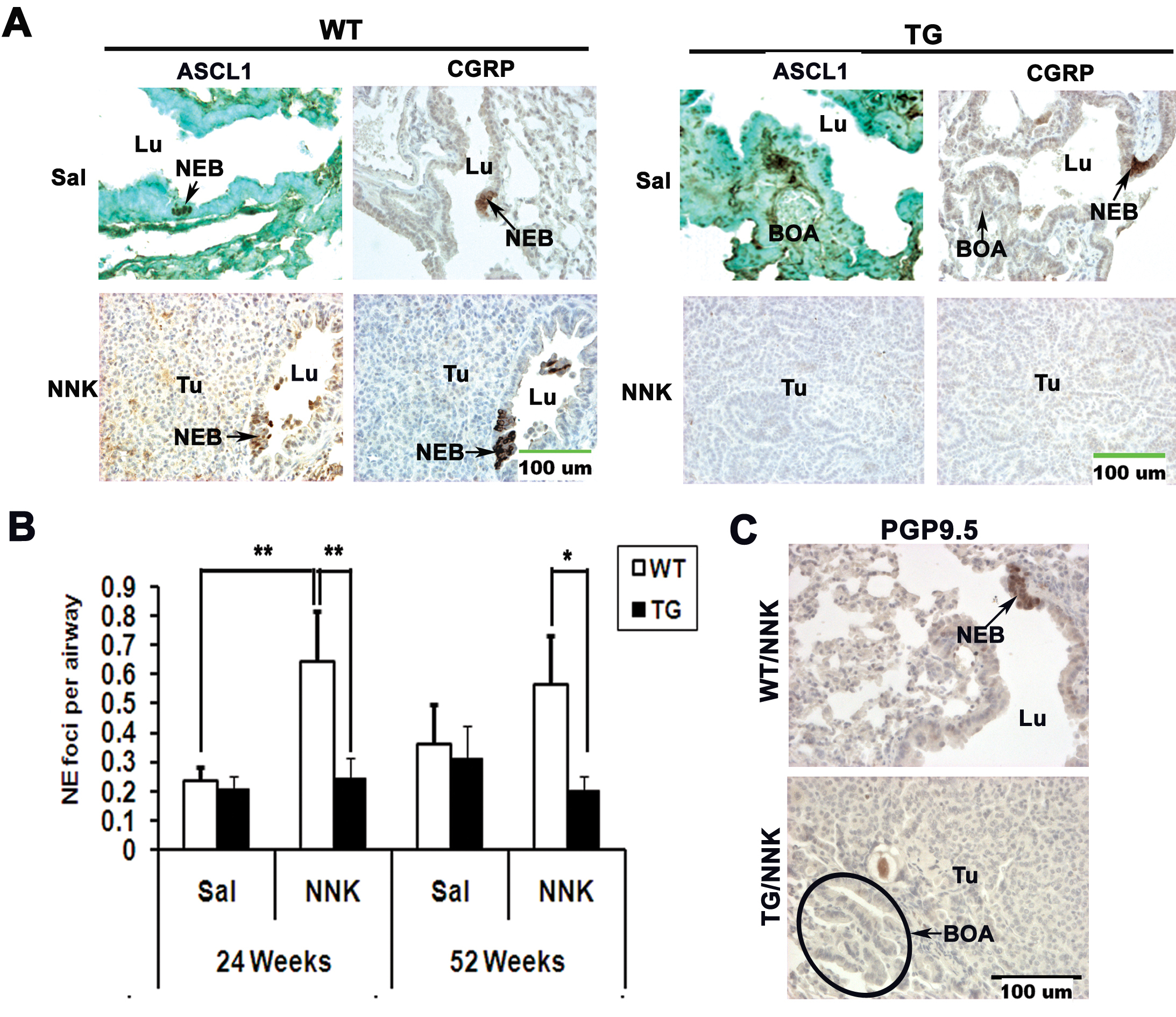

Supplement: Figure S2 — Constitutive expression of Ascl1 in Clara cells attenuates NNK-induced neuroendocrine (NE) cell differentiation in TG mice. A) Expression of Ascl1 and CGRP in small clusters (NEBs) of NE cells (arrows) in WT mice, surrounded by negative airway epithelium and negative tumors (left panels). In TG mice, Ascl1 was expressed throughout airway epithelium and BOAs, while the NE marker CGRP was expressed in NE cells (NEB with arrow). Airway epithelium and BOA (BOA with arrow) were negative for CGRP. No Ascl1 or CGRP expression was present in tumors (bottom panels; immunoperoxidase staining). B) Bar graph of the relative numbers of CGRP-containing neuroendocrine (NE) foci (number of solitary cells plus NEBs per airways; mean±S.E.M. * P<0.05, ** p<0.01, *** p<0.001). NNK exposure increased the number of NE foci in the airways 24 weeks in WT mice, but not in TG mice. At 52 weeks, NE foci remained fewer in TG mice than in WT mice with NNK treatment. WT = wild type mice, TG = transgenic mice, Sal = saline. * p<0.05, *** p<0.001. C) A PGP9.5 positive NEB in a WT/NNK (arrow, top panel). No PGP9.5 staining in BOA (ellipse with arrow) or tumor of TG/NNK mice (bottom panel) (immunoperoxidse staining). Lu = airway lumen, Tu = tumor, BOA = bronchiolization of the alveoli, NEB = neuroepithelial body. (JPG) [file pone.0052832.s002.jpg]

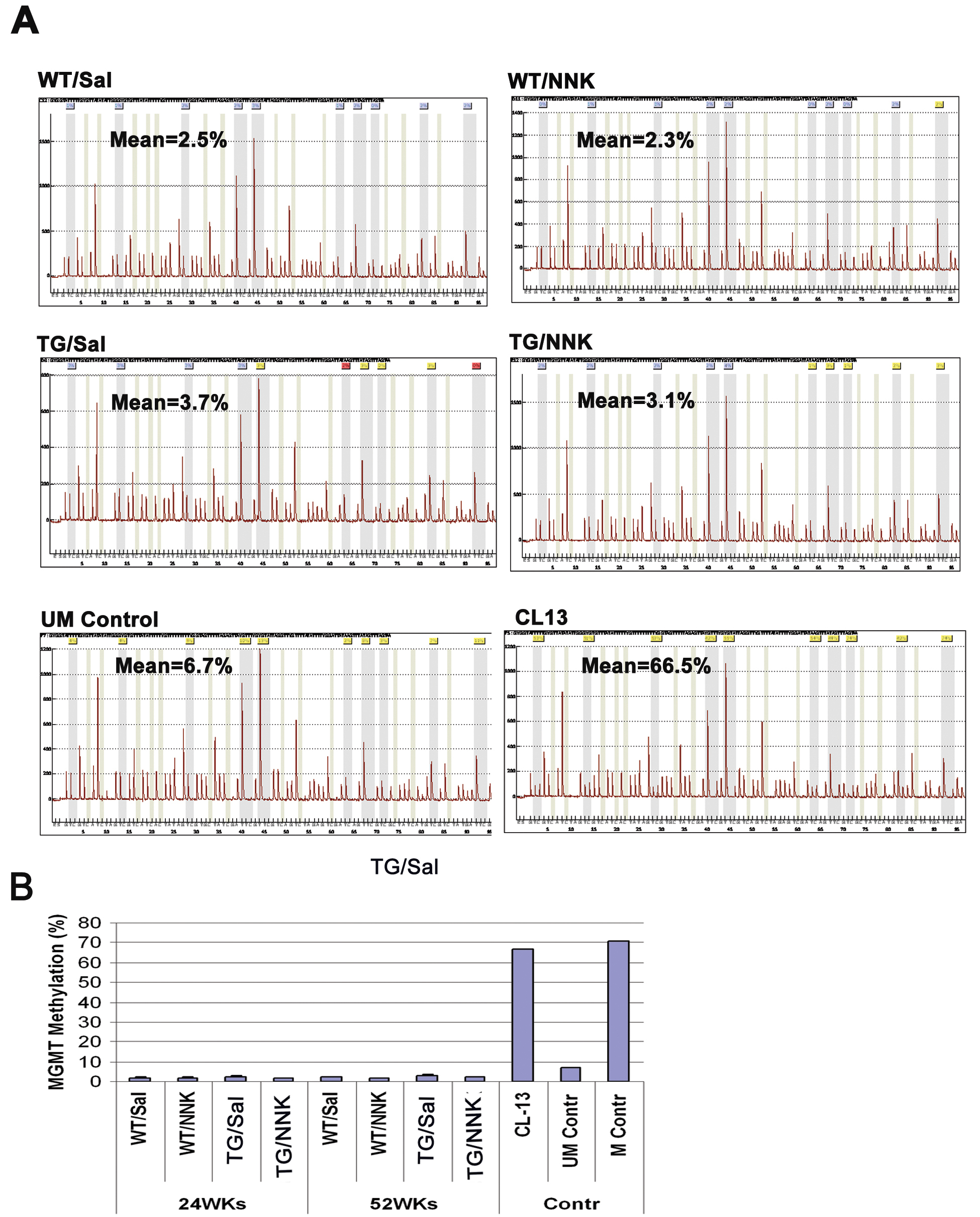

Supplement: Figure S3 — Lack of MGMT methylation in the lung detected by pyrosequencing. A) Representative pyrograms of MGMT pyrosequencing results of lungs from WT and TG mice with or without NNK treatment. Bottom two panels include a negative (UM = unmethylated) control and methylated mouse lung cancer cell line (CL-13) as a positivecontrol. B) MGMT promoter in mouse lung cancer cells CL-13 (positive control) showed methylation in 66.5% of the CpG islands, while no methylation was found in lung tissues. (JPG) [file pone.0052832.s003.jpg]
