# Supplementary material for: Achaete-Scute Complex Homolog-1 Promotes DNA Repair in the Lung Carcinogenesis through Matrix Metalloproteinase-7 and O(6)-Methylguanine-DNA Methyltransferase
Source: PLoS One. 2012 Dec 26;7(12):e52832. doi: 10.1371/journal.pone.0052832 (PMC3530493; doi:10.1371/journal.pone.0052832)
Supplement: Table S1 — Sequences of the oligonucleotide primers. (DOC) [file pone.0052832.s004.doc]

**Supporting Information**

**Table S**1. Sequences of the oligonucleotide primers

| **For quantitative RT-PCR** |
| --- |
| Sequences of the oligonucleotide (Genebank Access Number) |
| **Mouse MMP-7 (NM_010810 )** |
| Forward: 5’-TAGGCGGAGATGCTCACTTT-3' |
| Reverse: 5’-TTCTGAATGCCTGCAATGTC-3' |
| **Human MMP-7 (BC003635)** |
| Forward: 5’-TGGTAGCAGTCTAGGGATTAACTTCCT-3' |
| Reverse: 5’-CATAGGTTGGATACATCACTGCATTA-3' |
| **Mouse MGMT (NM-008598)** |
| Forward: 5'-AGATGGAGCTGTCTGGCTGTG-3' |
| Reverse: 5’-CCTCTGTGGGGTCAGTGTTTG-3' |
| **Human MGMT (NM_002412)** |
| Forward: 5'-gcaatgagaggcaatcctgt-3' |
| Reverse: 5'-cagtcctccggagtagttgc-3' |
| **Human Ascl1 (NM-004316)** |
| Forward: 5'-TCCCCCAACTACTCCAACGAC-3' |
| Reverse: 5'-CCCTCCCAACGCCACTG-3' |
| **18S rRNA (NR_003278 )** |
| Forward: 5'-TCGGAACTGAGGCCATGATT-3' |
| Reverse: 5'- CCTCCGACTTTCGTTCTTGATT-3' |
|  |
| **For ChIP assay** |
| **MMP-7** |
| Forward: 5’-AGAGAGTCTCCCTCTGTCAC-3’  Reverse: 5’-CTGAGGCACGAGAACTGCTTG-3’ |
| **MGMT** |
| Forward: 5’-TGGCATAGGTGCTGAGTTGA-3 |
| Reverse: 5’-TCCCGAAGACAAACTGTTCA-3’ |

Abbreviations: RT-PCR= reverse transcriptase PCR, ChIP= chromatin immunoprecipitation
