# Supplementary material for: Achaete-Scute Complex Homolog-1 Promotes DNA Repair in the Lung Carcinogenesis through Matrix Metalloproteinase-7 and O(6)-Methylguanine-DNA Methyltransferase
Source: PLoS One. 2012 Dec 26;7(12):e52832. doi: 10.1371/journal.pone.0052832 (PMC3530493; doi:10.1371/journal.pone.0052832)
Supplement: Table S2 — List of Antibodies for Immunohistochemistry and Western blot. (DOC) [file pone.0052832.s005.doc]

**Supporting Information**

**Table S2**. List of Antibodies for Immunohistochemistry and Western blot

| Antibody | Source | Dilution | | Positive control |
| --- | --- | --- | --- | --- |
|  |  | IHC | WB |  |
| Ascl1 (M) | BD BioSciences | 1:50 | 1:500 | Lung NE |
| CC10 (P) | Dr. F.J. DeMayo | 1:100,000 |  | Normal airway |
| Pro-SPC(P) | Millipore | 1:2000 |  | Type II cells |
| cGRP(P) | Sigma | 1:3000 | 1:1000 | Lung NE |
| PGP9.5 (P) | AbD Serotec | 1:6000 |  | Lung NE |
| MGMT(M) | Millipore |  | 1:300 | BEAS-2B/MMP7 cells |
| MGMT (FL-207) (P) | Santa Cruz | 1:200 |  | Normal airway |
| ß-Actin (P) | Cell Signaling |  | 1:1000 | human cells |
| MMP-7 (P) | Dr. B. Fingleton |  | 1:2000 | Lung cancer |
| P-H2Ax (P) | Cell Signaling |  | 1:1000 | H_2_O treated cells |

Abbreviations: M=monoclonal, P=polyclonal, IHC= Immunohistochemistry, WB= Western blot, NE= neuroendocrine cells
